# COMBINED EFFECT OF CMV SEROPOSITIVITY AND SYSTEMIC INFLAMMATION ON DEMENTIA PREVALENCE IN CANCER SURVIVORS

**DOI:** 10.1093/geroni/igz038.1724

**Published:** 2019-11-08

**Authors:** Sithara Vivek, Bharat Thyagarajan, Heather Nelson, Anna Prizment, Eileen Crimmins, Jessica Faul

**Affiliations:** 1 University of Minnesota, Minneapolis, Minnesota, United States; 2 Department of Laboratory Medicine and Pathology University of Minnesota; Minneapolis, Minnesota, United States; 3 Davis School of Gerontology, University of Southern California, Los Angeles, California, United States; 4 University of Michigan, Ann Arbor, Michigan, United States

## Abstract

Though cancer patients treated with multi-modal therapies demonstrate higher levels of systemic inflammation, which is associated with dementia, cancer survivors have not shown a consistent association with dementia. Since several studies reported an independent association between cytomegalovirus (CMV) infection, inflammation and dementia in non-cancer populations, we have evaluated whether CMV infection and systemic inflammation were associated with increased prevalence of dementia in cancer survivors in Health and Retirement Study (HRS). We evaluated prevalence of dementia (using score ≤7 on the 27-point scale) among 1607 cancer survivors, in whom we measured CMV seropositivity and two biomarkers of systemic inflammation: C-reactive protein (CRP) and neutrophil-lymphocyte ratio (NLR). The prevalence of CMV seropositivity was 68.26% (n=1097), while prevalence of increased systemic inflammation [CRP >5mg/L and NLR >4] was 4.23% (n=68). Using survey logistic regression, adjusted for age, race, gender, BMI (Body Mass Index) and sampling design, cancer survivors who were both CMV seropositive and had increased systemic inflammation had the highest odds of dementia compared to those who were CMV seronegative and had low levels of systemic inflammation (OR=6.59; 95% CI [2.81, 15.44]; p<.0001). Cancer survivors who were CMV seropositive without evidence of systemic inflammation had a lower but increased odds of dementia (OR=2.02; 95% CI [1.17, 3.47]; p=0.01). Odds of dementia among those who were CMV seronegative with elevated systemic inflammation was not significant (p=0.09). Our study demonstrates a possible role for ongoing CMV induced inflammation in determining dementia prevalence among cancer survivors that needs further confirmation.

